# Facial shape differences between rats selected for tame and aggressive behaviors

**DOI:** 10.1371/journal.pone.0175043

**Published:** 2017-04-03

**Authors:** Nandini Singh, Frank W. Albert, Irina Plyusnina, Lyudmila Trut, Svante Pӓӓbo, Katerina Harvati

**Affiliations:** 1 Paleoanthropology, Senckenberg Center for Human Evolution & Paleoenvironment, Eberhard Karls Universität Tübingen, Tübingen, Germany; 2 Department of Anthropology, Pennsylvania State University, University Park, United States of America; 3 Max Planck Institute for Evolutionary Anthropology, Leipzig, Germany; 4 Department of Genetics, Cell Biology, and Development, College of Biological Sciences, University of Minnesota, Minneapolis, United States of America; 5 Institute of Cytology and Genetics, Siberian Branch of the Russian Academy of Sciences, Novosibirsk, Russia; 6 DFG Centre for Advanced Studies: Words, Bones, Genes, Tools: Tracking linguistic, cultural and biological trajectories of the human past, Eberhard Karls Universität Tübingen, Tübingen, Germany; University of Lethbridge, CANADA

## Abstract

Domestication has been consistently accompanied by a suite of traits called the domestication syndrome. These include increased docility, changes in coat coloration, prolonged juvenile behaviors, modified function of adrenal glands and reduced craniofacial dimensions. Wilkins et al recently proposed that the mechanistic factor underlying traits that encompass the domestication syndrome was altered neural crest cell (NCC) development. NCC form the precursors to a large number of tissue types including pigment cells, adrenal glands, teeth and the bones of the face. The hypothesis that deficits in NCC development can account for the domestication syndrome was partly based on the outcomes of Dmitri Belyaev’s domestication experiments initially conducted on silver foxes. After generations of selecting for tameness, the foxes displayed phenotypes observed in domesticated species. Belyaev also had a colony of rats selected over 64 generations for either tameness or defensive aggression towards humans. Here we focus on the facial morphology of Belyaev’s tame, ‘domesticated’ rats to test whether: 1) tameness in rats causes craniofacial changes similar to those observed in the foxes; 2) facial shape, i.e. NCC-derived region, is distinct in the tame and aggressive rats. We used computed-tomography scans of rat skulls and landmark-based geometric morphometrics to quantify and analyze the facial skeleton. We found facial shape differences between the tame and aggressive rats that were independent of size and which mirrored changes seen in domesticated animals compared to their wild counterparts. However, there was no evidence of reduced sexual dimorphism in the face of the tame rats. This indicates that not all morphological changes in NCC-derived regions in the rats follow the *pattern of shape change* reported in domesticated animals or the silver foxes. Thus, certain phenotypic trends that are part of the domestication syndrome might not be consistently present in all experimental animal models.

## Introduction

The classic selection experiments by Dmitry Belyaev have demonstrated that breeding animals for tameness can lead to a suite of physiological, cognitive and morphological changes similar to those associated with domestication [1,2]. Key morphological traits that comprise the domestication syndrome include changes in adreno-cortical responses, coat coloration, brain size, body size and craniofacial size and shape [3–5]. According to Belyaev, behavior–specifically tameness–was the driving factor behind all the traits present in domesticated animals [2]. Selective breeding of animals allows for a closer look at the initial factors that led to domestication. Behavior is regulated by neurotransmitters and hormones, which when altered by strict selection for a particular trait, can cause changes in key developmental processes, thereby potentially affecting the phenotype [6].

Physiological and genetic changes associated with domestication have been extensively documented in silver foxes (*Vulpes vulpes*) and rats (*Rattus norvegicus*) [4,7,8]. Selection for a behavioral trait such as tameness can result in ‘off-target’/unintentional changes in integrated developmental mechanisms[9]. Elaborating on this developmental perspective, Wilkins et al. [10,9] recently emphasized the connection between neural crest cell (NCC) development and certain key physiological and phenotypic features that comprise the domestication syndrome. According to Wilkins et al. [10], selection for tameness and reduced aggression towards humans led to alterations in NCC proliferation and migration, directly or indirectly causing the majority of the traits associated with domestication. NCC regulate and control bone and cartilage formation and several autonomous molecular and cellular processes. A number of physiological and phenotypic structures altered in domesticated animals are derived from NCC. Of particular interest to this study is increased morphological variation and phenotypic novelty present in the facial structures of animals selected for tameness and aggression, and how those changes related to domesticated animals compared to their wild counterparts. Across vertebrates, the NCC gives rise to the entire facial skeleton, anterior aspects of the cranial base and frontal bones from NCC across vertebrates [11–13].

In this study we focus on Belyaev’s tame and aggressive rat colonies. Since 1972, these animals have been selectively bred in two populations for either tame or aggressive behaviors towards humans [1,5,6]. Daughter colonies of the tame and aggressive rats were established in Leipzig, Germany in 2005 [4,7]. The tame rats show substantially reduced defensive tendencies towards humans whereas the aggressive line shows overt defensive aggression towards their handlers [3,14]. The tame rats also presented changes in coat color variation not seen in the aggressive strain, but similar to that observed in the tame foxes [4,15]. Likewise, domesticated dogs, pigs and horses often exhibit coat color variation not present in their wild counterparts. These phenotypic changes suggest a potential link between selection for certain ‘domesticated’ behaviors and morphology [9,16,17].

Here we use the experimental rat model for animal domestication to examine the relationship between behavior and craniofacial shape change. In doing so, we evaluate whether tameness and aggression causes shape changes in NCC-derived region, such as the face, and whether those changes mimic the pattern observed in the silver foxes. While several studies have looked at the effects of tameness and aggression on the genetic architecture of these rats [4,5,8,14], a quantitative study, mapping the precise patterns of facial shape change has not been thus far conducted on this sample. Following Belyaev’s original premise that tameness was the precursor to most domesticated traits, our hypothesis is that the tame rats would exhibit similar *pattern* of phenotypic changes observed in the silver foxes and other domesticates. In this regard, such animal models are particularly useful for studying how selection on one trait or a shift in one aspect of behavior can produce multiple unselected morphological traits. They can further be used to investigate developmental mechanisms that underlie the domestication syndrome.

## Materials and methods

The animals we studied here are the same animals described in the original characterization of the Leipzig populations of these rats [4]. Briefly, the rats that had been brought to Leipzig were from the 64^th^ generation of selection. The selection regime in Novosibirsk entailed testing the reaction of these rats to humans, measured through the rats’ reaction to an approaching hand. The animals studied here are from the 2^nd^ and 3^rd^ generation born in Leipzig. All animals were maintained in identical cages and conditions, and treated exactly the same way, including the level of interaction with the human handlers. Animals were weighed, anaesthetized with CO_2_ and killed by cervical dislocation. Further details on the experimental regime and maintenance of the rat colonies can be obtained in Albert et al. [4]. All procedures were reviewed and approved by the regional government of Saxony (TVV 29/05), Germany.

**Fig 1:**
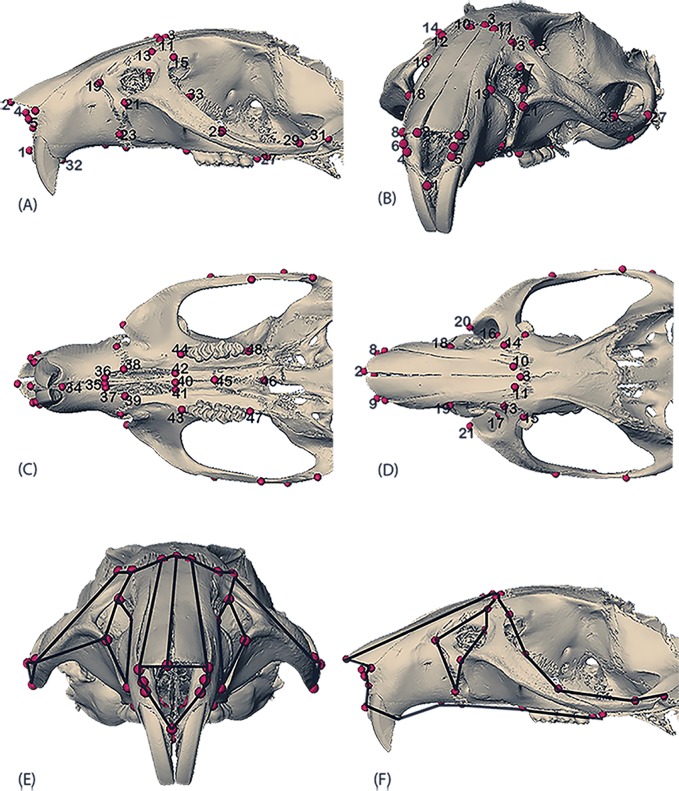
Landmarks used in the study. (A) Rostral view; (B) Diagonal view; (C) Lateral view; (D) Ventral view; (E) Doral view with wireframe; (F) Lateral view with wireframe. Definition of landmarks corresponding to the number in Table 2.

High-resolution micro-computed tomography images were acquired for fifty-five adult rat skulls (Table 1) at the University of Tübingen (Paleoanthropology High Resolution CT Laboratory) with 0.05mm resolution along the x, y, z axes for each 16bit rat image. For each scan, isosurfaces were created for landmark data collection using the software package Avizo 6.3 (Visualization Sciences Group, VSG) and forty-eight three-dimensional cranial landmarks (Fig 1, Table 2) were measured on them by NS. Intra-observer error tests were conducted by re-measuring ten individuals a year later. Measurement error was negligible, with the difference between the two trials < 0.05mm.

**Table 1:** Sample used in the study.

Strains	Numbers	Females	Males	Species
Tame	33	17	16	*Rattus norvegicus*
Aggressive	22	11	11	*Rattus norvegicus*
**Total**	**55**	**28**	**27**	

**Table 2:** Landmarks used in this study.

Landmark Numbers	Landmark Definitions
1	Midpoint, intersection of the anterior alveolar region
2	Midpoint, anterior point between the nasal bones
3	Midpoint, intersection of the nasal bones
4&5	Inferior lateral points on the nasal aperture
6&7	Lateral and superior points on the nasal aperture
8&9	Lateral points on the intersection of the nasal bones and premaxillae
10&11	Lateral points on the nasal ones
12&13	Anterior most points on maxillary-frontal suture
14&15	Posterior points on maxillary-frontal suture
16&17	Superior most point on the infraorbital foramen
18&19	Anterior most points on the premix-maxillary suture (in front of infraorbital foramen
20&21	Lateral most points on the infraorbital foramen
22&23	Inferior most points on the premax-maxillary suture
24&25	Superior most point on the zygomatic suture (anterior)
26&27	Inferior most point on the zygomatic suture (posterior)
28&29	Superior most point on the zygomatico-temporal suture
30&31	Inferior most point on the zygomatico-termporal suture (posterior)
32&33	Anterior most point (behind the infraorbital foramen) on the maxillary-frontal suture
34	Midpoint, posterior of the incisors
35	Midpoint, between the palatine foramen
36&37	Anterior most points on the palatine foramen
38&39	Lateral most point on the palatine foramen (at the suture)
40	Midpoint, posterior point on the palatine suture
41&42	Posterior most points on the palatine foramen
43&44	Anterior most points on the tooth row
45	Midpoint, anterior intersection of the palatine bones
46	Midpoint, posterior intersection of the palatine bones
47&48	Posterior most points on the tooth row

Generalized Procrustes Analysis (GPA) was used to superimpose the forty-eight cranial landmarks (Table 2). This method extracts superimposed Procrustes coordinates from the original landmark data by translating, scaling and rotating the original coordinates and subsequently yielding a measure for size called Centroid size (defined as the square root of the summed squared deviations of the landmark coordinates from their centroid [18,19]. A Principal Components Analysis (PCA) of the Procrustes coordinates was performed to examine the overall variation in the dataset. PCA is based on an eigenvalue decomposition of a covariance matrix and distributes the shape variation as scores along the different PCs [19]. The facial shape variation along the PC axes was visualized on surface scans and wireframe diagrams. The surface warps were generated in Avizo 6.3 by warping the PC scores onto the mean shape of all the specimens in the sample, and the wireframe diagrams were constructed in MorphoJ [20]. The allometric variation, i.e. size related shape differences, was examined through a multivariate regression of Procrustes shape coordinates on centroid size. An additional PCA was conducted on the multivariate regression residuals (of shape on size) to examine the overall shape variation in the data without the effects of allometry. All analyses were conducted in the programming software R version 3.1.2 (The R FAQ http://cran.r.project.org/doc/FAQ/R-FAQ.html) and MorphoJ.

## Results and discussion

### Behavior and facial shape variation

**Fig 2:**
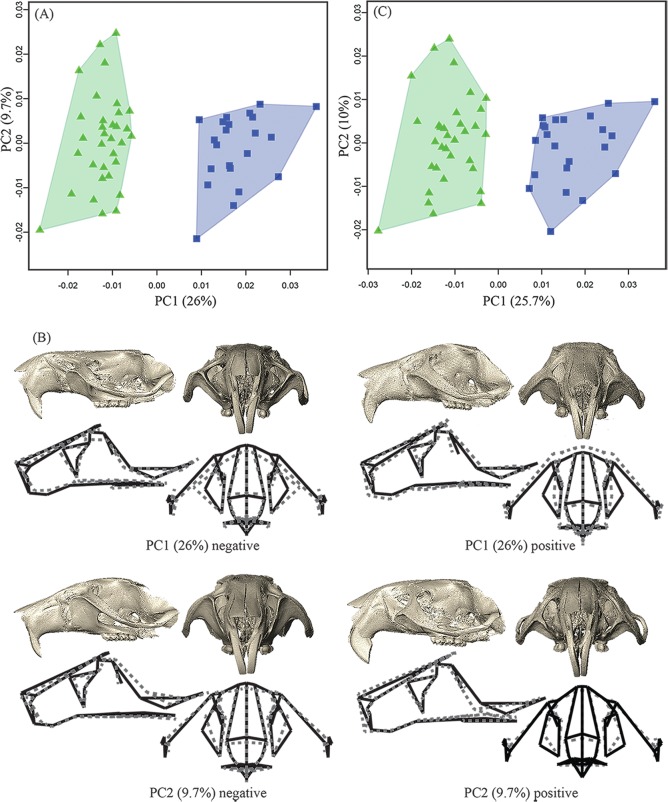
PCA plots of the overall variation in the dataset. (A) PCA plot in shape-space showing a distinct separation between the tame (green triangles) and aggressive (blue squares) groups along PC1 and within group variation along PC 2; (B) Surface morphs and wireframes depicting the shape changes along the scores of PC1 and PC2 in Fig 1(A). The surface morphs are computed by warping the respective PC scores onto the mean shape of all the specimens in the sample and illustrate the shape changes from the negative to the positive end of the PC axes. The same shape changes are also represented by the wireframe diagrams, the dotted lines showing the changes along each PC axis against the mean shape in solid lines, of the sample. PC1 captures shape changes, primarily in the face, between tame (green triangles) and aggressive (blue squares) rats. The tame rats in green triangles (on the negative end of PC1) show retraction in the anterior aspects of the snout and dorso-ventral contraction of the anterior cranial vault, compared to the aggressive rats in blue squares (on the positive end of PC1); PC2 accounts for the within-group and shared shape changes in the two groups, capturing variation in the position of the incisors and lateral aspects of the infraorbital foramen; (C) PCA on regression residuals showing little change between and within the groups when size is regressed out from the analysis.

Results of the PCA show a clear distinction in facial morphology between the tame (n = 33) and aggressive (n = 22) strains of rats (Table 1) on principal component 1 (PC1, 26% of total variance; Fig 2A). Within-group variation is similar in the two strains, with the tame sample exhibiting a slightly larger range of variation than the aggressive one along PC2 (9.7% of total variance; Fig 2A). The separation along PC1 reflects shape differences in the medio-lateral and dorso-ventral facial dimensions (Fig 2B). Compared to the aggressive group, the tame sample shows a slightly dorso-ventrally contracted and medio-laterally expanded face (marked by the lateral placement of the infraorbital foramen) and a retracted snout (indicated by inferior aspect) and supero-inferiorly reduced snout height, along the negative scores on PC1 (Fig 2B). Both groups vary similarly along PC2 (Fig 2A), primarily showing changes in the height and curvature of the snout (marked by the placement of the nasal bones) and rotation (upwards on the negative scores and downwards on the positive end) of the infraorbital foramen, maxillary and zygomatic bones (Fig 2B). Importantly, when the PCA was performed on multivariate regression residuals, in order to reduce the effects of allometry, the group scatters remain almost identical (Fig 2C). These results indicate that facial shape changes between these groups are independent of size-related differences.

The morphological features that distinguish the tame rats from the aggressive rats parallel some of the phenotypic changes associated with other experimental and intentionally domesticated animals. Particularly, the overall medio-lateral expansion, dorso-ventral contraction and retraction in some aspects of the snout, resembles features noted in the tame silver foxes, the first animals used in Belyaev’s selection experiments [5,8,21]. Specifically, the tame foxes had superior-inferiorly smaller crania (i.e. cranial height), and wider and shorter snouts compared to their control counterparts [2,8], but these changes were more pronounced in the tame males than the tamefemales [8], a trait not shared with the rats. Similar modifications in cranial morphology were also noted in domesticated dogs relative to wolves [8,21–24]. These results suggest a potential link between behaviors and specific craniofacial changes, some of which are shared among experimental animals, like shortening and widening of the snout.

### Sexual dimorphism

**Fig 3:**
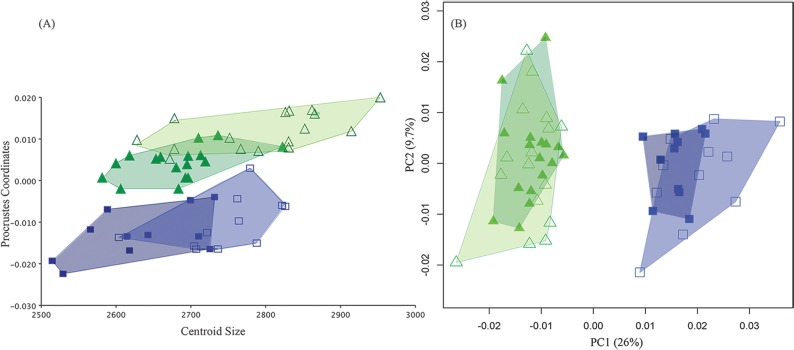
Quantitative analyses of sexual dimorphism in the dataset. (A) Plot of multivariate regression of Procrustes shape coordinates on Centroid size showing differences between males (open green triangles and open blue squares in the respective tame and aggressive groups) and females (solid triangles and solid squares in the respective tame and aggressive groups) in the tame (green triangles) and aggressive (blue squares) rats; (B) PCA plot showing similar shape variation between the sexes in the two groups.

In domesticated species, reduced aggression and tolerance of humans is often accompanied by reduced sexual dimorphism as measured by reduction in overall cranial size and shortened snouts. These traits are part of the anatomical changes that constitute the domestication syndrome [24]. Although there has been no previous comparative work on the cranial morphology of rats selected for tameness, earlier studies on silver foxes reported a decrease in craniofacial proportions of the tame male foxes, where the overall skull of the tame males was observed to be more ‘feminized’ (i.e. reduction in size) compared to their aggressive counterparts, displaying reduced sexual dimorphism [8,25]. Following this, we examined the pattern of sexual dimorphism in the tame and aggressive rats to assess whether selection on tameness ‘feminized’ the male rat cranial morphology in terms of showing a reduction in *size*. A multivariate regression analysis of Procrustes shape variables on size revealed the tame male rats to be larger than the tame females (Fig 3A), suggesting a degree of sexual dimorphism. In fact, the tame males were the largest group in our sample, lacking the cranial size reduction reported for the tame male foxes and males of other domesticated species. This pattern of sexual size dimorphism was also seen in the aggressive strain (Fig 3), with the males being slightly bigger than the females. By contrast, neither strain showed sexual dimorphism in *shape*. Males and females showed no distinction in aspects of shape despite being dimorphic in cranial size (Fig 3B) in either group. Thus, the size difference between sexes cannot be explained by differences in facial shape.

The differences in facial size between females and males are not associated with differences in behavior as both sexes displayed the same level of tameness or defensive aggression towards their human handlers [4,26]. Instead, the pattern seen in our dataset suggests that the ‘normal’ size dimorphism found in wild Norway rats, where the males are bigger than the females in body size and weight, was maintained in the two groups despite years of selective breeding for specific behaviors [27]. The hypothesis of reduced sexual dimorphism in tame animals would have predicted a pattern of size sexual dimorphism comparable to that reported for the foxes, where the tame groups showed an overall reduction in cranial size and sexual dimorphism compared to the aggressive animals. However, here the absence of a reduction in overall facial size of the tame rats shows that experimentally domesticated animals do not necessarily share all phenotypic patterns of change described in the domestication syndrome.

### Role of behavior in evolutionary change: Developmental perspective

Both the tame foxes and the tame rats displayed behaviors more associated with juvenile animals, showing excessive friendliness, reduced stress and increased affinity towards humans. Domesticated animals often display juvenile-like behaviors and exhibit physical traits that are considered neotenous compared to their wild ancestors. Paedomorphism and other socio-cognitive changes in domesticated species, particularly dogs [28–31], have been attributed to selection against aggressive behaviors. However, Drake [32] showed that dogs are not paedomorphic wolves in their craniofacial morphology despite exhibiting an array of morphological traits that are part of the domestication syndrome. Drake [32] found that present day dog breeds did not have neomorphic crania compared to their wolf ancestors and did not resemble juvenile wolves. Drake’s [32] study brings into question whether paedomorphism in craniofacial dimensions can be considered a consistent trait across all domesticated species.

Nonetheless, because behavioral responses are regulated by a number of hierarchical and integrated developmental networks, such as the interactions between neurotransmitters and hormones [33–37], it is likely that rigorous selection for certain behaviors will affect other aspects of the genotype, consequently affecting the phenotype [38]. In this regard, experimental models for domestication can provide ways to test the role of NCC and other developmental pathways in generating traits most commonly found in domesticated animals. For example, perturbations in the sonic hedgehog pathway [39–43], fibroblast growth factors and their respective receptors [44–46] and bone morphogenetic proteins [47–49] affect growth and development of the craniofacial form, but the role of NCC in regulating these important developmental networks still needs further investigation [50].

Changes in craniofacial morphology, particularly in aspects of the midface/snout, as seen in both the aggressive and tame rats, could potentially be expressing differences based on the underlying molecular changes that are regulated by NCC. Breeding experiments selecting for specific behaviors can further our understanding of how behavior influences the underlying developmental-genetic networks that give rise to the traits outlined in the domestication syndrome [9,16]. These experimental systems might also provide evidence that refutes the notion that the domestication syndrome is a direct consequence of tameness alone. Clearly from our results and from evidence from dog domestication [32] not all features of the domestication syndrome are consistently present in all domesticated animals.

## Conclusions

Animal and plant domestication has been a focus of intense research at least since the time of Darwin [51]. Experimental animal models have added a new dimension to archaeological evidence by providing novel ways in which we can address a number of questions regarding the genetic and developmental origins of animal domestication. Our findings show that targeted selection for reduced aggression in Belyaev’s rats is accompanied by changes in the face, which is entirely derived from NCC, and that those changes are independent of cranial size. This result suggests that the craniofacial changes in the tame rats might be a developmental-genetic ‘side effect’ of selection for tameness. However, the tame rats do not perfectly mimic domesticated animals in that they do not show a notable reduction in facial sexual dimorphism.

The rat model for domestication provides important nuance to our understanding of the evolution of cranial morphology in domesticated species in that they demonstrate that not all the phenotypic traits outlined in the domestication syndrome are necessarily always present when selecting for tame behavior. Even though our study focuses on the morphological consequences of selecting for specific behaviors, it highlights the need to further investigate how the evolution of behavior can affect the evolution of other phenotypes and, more importantly, whether or not other NCC-derived regions that are part of the domestication syndrome show consistent and comparable changes across domesticated species.
